# Design of A High-Precision Component-Type Vertical Pendulum Tiltmeter Based on FPGA

**DOI:** 10.3390/s23187998

**Published:** 2023-09-20

**Authors:** Xin Xu, Zheng Chen, Hong Li, Shigui Ma, Liheng Wu, Wenbo Wang, Yunkai Dong, Weiwei Zhan

**Affiliations:** 1 National Institute of Natural Hazards, Beijing 100085, China; xuxin212@mails.ucas.ac.cn (X.X.);; 2 School of Emergency Management Science and Engineering, University of Chinese Academy of Sciences, Beijing 100049, China

**Keywords:** tiltmeter, digital signal processing, FPGA

## Abstract

This paper presents a high-precision component-type vertical pendulum tiltmeter based on an FPGA (Field Programmable Gate Array) that improves the utility and reliability of geophysical field tilt observation instruments. The system is designed for rapid deployment and offers flexible and efficient adaptability. It comprises a pendulum body, a triangular platform, a locking motor and sealing cover, a ratiometric measurement bridge, a high-speed ADC, and an FPGA embedded system. The pendulum body is a plumb-bob-type single-suspension wire vertical pendulum capable of measuring ground tilt in two orthogonal directions simultaneously. It is installed on a triangular platform, sealed as a whole, and equipped with a locking motor to withstand a free-fall impact of 2 m. The system utilizes a differential capacitance ratio bridge in the measurement circuit, replacing analog circuits with high-speed AD sampling and FPGA digital signal processing technology. This approach reduces hardware expenses and interferences from active devices. The system also features online compilation functionality for flexible measurement parameter settings, high reliability, ease of use, and rapid deployment without the need for professional technical personnel. The proposed tiltmeter holds significant importance for further research in geophysics.

## 1. Introduction

A high-sensitivity tilt survey serves as a crucial method for crustal deformation observation and plays a vital role in geodetic, seismological, and geophysical research. In recent years, geoscientific research has placed great emphasis on geotilt observations, such as Plate Boundary Observations (PBO), an important component of the Earth Lens Program implemented in the United States and the China Seismic Proving Ground Project, which have deployed numerous tilt observation instruments [1,2,3]. Traditional geophysical field tilt observation instruments, including horizontal pendulum tiltmeters, vertical pendulum tiltmeters, and water pipe tiltmeters, offer a sensitivity of 0.2 mas (milliarcseconds), enabling the observation of solid earth tides, small crustal deformation, and co-seismic responses [4,5,6,7,8]. However, these instruments, being delicate and lacking impact resistance and reliability, require installation and operation by skilled engineering technicians. This limitation hinders widespread and rapid deployment.

**Figure 1:**
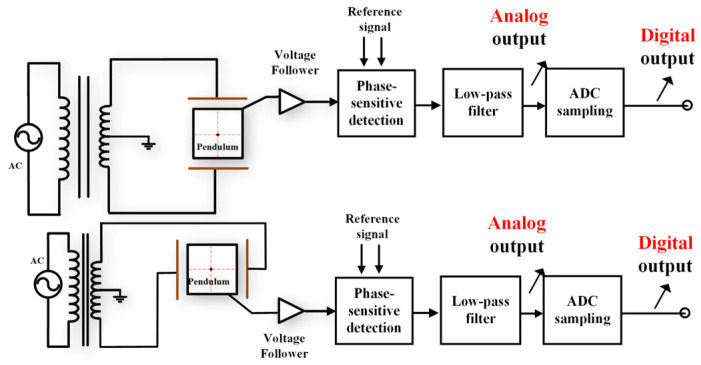
Vertical pendulum inclinometer structure and measurement circuit diagram.

In the traditional vertical pendulum tilt meter detection circuit shown in Figure 1, the circuit is mainly AC excitation, transformer, differential capacitance sensor, voltage follower, phase sensitive detector, low-pass filtering circuit, etc. The direction of the pendulum is always vertically downward but with the change of tilt angle, the pole–plate spacing changes to change the capacitance. The transformer and differential capacitance sensor form an AC bridge, and as the capacitance changes, the unbalanced signal output from the bridge circuit is output to the ADC acquisition unit through a voltage follower, phase-sensitive detector, and low-pass filtering. Since the tilt observation requires two components, north–south and east–west, and two sets of the same sensors and circuits are required to accomplish this.

Figure 1 is a classic and widely used tilt measurement circuit, but with the deepening of geophysical research and the increasing requirements of tilt observation, the limitations of the above measurement circuit have gradually appeared.

First, tilt measurement requires two sets of vertical pendulums and detection circuits, making it difficult to achieve an integrated design. Secondly, the phase-sensitive detector and low-pass filter circuits are built by analog devices, which inevitably introduce electrical noise, which makes the measurement accuracy of the circuits limited by the performance of the devices. Parameters such as frequency band, amplification, phase angle, etc., of the measurement system are all determined by analog devices and cannot be flexibly changed when used.

Currently, some scholars have integrated the measurements of north–south and east–west directions into a single sensor under laboratory conditions while utilizing FPGAs for signal processing to separate the two tilt signals. However, the excitation source still consists of analog circuits. After the measurement in one direction exceeds the limit, the data will be in error and cannot continue to complete accurate measurements [9].

This paper introduces an improved ICT (Integrated Capacitive Tiltmeter) integrated chamber tiltmeter, namely a high-precision component-type vertical pendulum tiltmeter based on an FPGA. FPGA compared to MCU and DSP, FPGA can combine the advantages of both, realizing the functions of both DSP and MCU, which is suitable for computing scenarios with fixed tasks. The system accomplishes simultaneous measurements in both directions with a single pendulum. The system measurement circuit employs a differential capacitance ratio bridge, where both bridges share the same pendulum as the intermediate electrode plate of the differential capacitor, facilitating the simultaneous measurement of ground tilt in two orthogonal components [1]. The system replaces analog circuits such as phase-sensitive detection, phase shifting, and low-pass filtering with high-speed AD sampling and FPGA digital signal processing technology [10]. This approach reduces hardware expenses and mitigates interference from active devices [11]. The online configuration function allows for flexible measurement parameter settings and adaptation to various measurement environments. At the same time, in the event of an abnormality or overrun of one of the signals, the FPGA stops the output of that channel to ensure that the measurement continues.

By leveraging the integrated design of the tilting pendulum system and FPGA digital signal processing technology, the improved ICT integrated chamber tiltmeter offers high reliability, strong adaptability, user convenience, and rapid deployment without the need for professional technical personnel. The instrument has high resolution and can accurately record solid tides, co-seismic response, etc., which is of great significance for geophysical field deformation observation.

## 2. Design of a High-Precision Tiltmeter Based on FPGA

**Figure 2:**
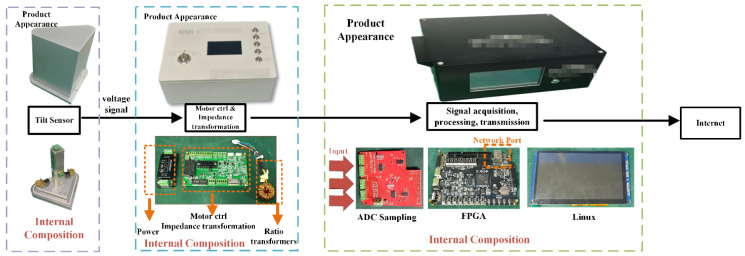
Schematic diagram of a high-precision tiltmeter system based on an FPGA.

The integrated high-precision chamber tiltmeter based on FPGA mainly consists of a vertical pendulum body, a triangular platform, a locking motor and sealing cover, a ratio-metric measurement bridge, a high-speed ADC, and an FPGA embedded system. Figure 2 illustrates the configuration of the vertical pendulum body, which serves as the tilt-sensing unit and is installed on a triangular platform. The platform is driven using micron-level controlled high-precision decelerated stepper motors, and the platform serves as both a tilt leveling mechanism and an online calibration of the tilt sensor [12,13,14,15]. The tilting pendulum and the adjusting platform adopt an integrated design; the whole is sealed and protected, and the pendulum is equipped with a locking motor. Due to the fragile hanging wire, it is necessary to push the pendulum to a certain side to fix it when it is not in working condition. The instrument is usually installed in caves—the environment is humid, so it needs to be sealed cover for protection and moisture to prevent short circuits. It can withstand a free fall impact of 2 m when it is in a locked and wrapped state. Tilt measurement is achieved through a ratio-metric bridge circuit. With the exception of the power amplifier of the excitation source, the output transformer, and the signal follower, the remaining circuitry is implemented through software programming after high-speed ADC signal sampling under FPGA control.

### 2.1. Component-Type Vertical Pendulum Tilt Sensing Unit

**Figure 3:**
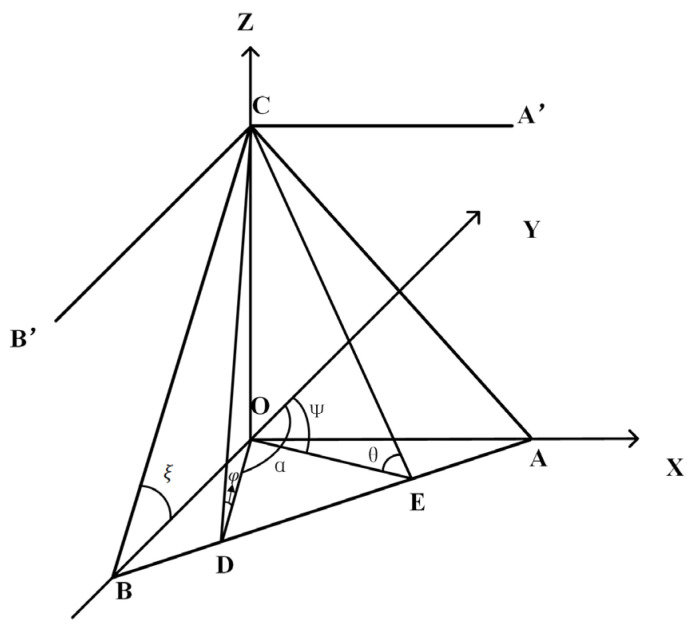
Schematic diagram of ground tilt.

Ground tilt follows the vector synthesis law only in two orthogonal directions. Figure 3 depicts the schematic diagram of ground tilt, where Z represents the gravity direction coinciding with the plumb line. The X and Y axes represent the east–west and north–south directions, respectively. The XOY plane denotes the horizontal plane, and ABC represents the tilting plane denoted as “t”. ∠CEO represents the dihedral angle between the tilting plane and the horizontal plane. The tilt angle of the tilting plane is denoted as θ = ∠CEO, and the azimuth angle of OE is denoted as *Ψ*. As ∠CEO represents the dihedral angle between the tilting plane and the horizontal plane, AB⊥OE, and AB⊥CE. AB represents the intersection line between the tilting plane and the horizontal plane, showing the strike of the tilting plane on the horizontal plane. θ and ψ are two characteristic parameters of the tilting plane denoted as “t”, representing the angle and direction of tilt, respectively [16,17,18].

In the figure, along the direction of azimuth α (OD direction), the measured tilt is φ. Then:

(1) φ=θcos⁡(α−ψ)

Assuming $\alpha_{1}$ and $\alpha_{2}$ are two different azimuth angles, and the ground tilt values in these two directions are $\varphi_{1}\;and\;\varphi_{2}$, respectively, and assuming positive tilt values in the $\alpha_{1}$ and $\alpha_{2}$ directions and negative values in the opposite directions, Equations (2) and (3) can be derived:

(2) $$\varphi_{1}=\theta \cos\left(\alpha_{1}-\psi \right)$$

(3) $$\varphi_{2}=\theta \cos\left(\alpha_{2}-\psi \right)$$

From Equations (2) and (3), θ and ψ can be obtained:

(4) $$\left\{\begin{array}{c} \theta =\frac{\sqrt{{\left(\varphi_{1}\cos\alpha_{2}-\varphi_{2}\cos\alpha_{1}\right)}^{2}+{\left(\varphi_{2}\sin\varphi_{1}-\varphi_{1}\sin\varphi_{2}\right)}^{2}}}{\left|\sin\left({\alpha_{2}-\alpha }_{1}\right)\right|}\\ \psi =\tan^{-1}\left(x/y\right) \end{array}\right.$$

where:

(5) $$\left\{\begin{array}{c} x=\frac{\varphi_{1}\cos\alpha_{2}-\varphi_{2}\cos\alpha_{1}}{\sin\left({\alpha_{1}-\alpha }_{2}\right)}\\ y=\frac{\varphi_{2}\sin\varphi_{1}-\varphi_{1}\sin\varphi_{2}}{\sin\left({\alpha_{1}-\alpha }_{2}\right)} \end{array}\right.$$

The quadrant where ψ is located is determined by the signs of *x* and *y*. When measuring the tilt values in the north–south and east–west directions, let ξ and η represent the ground tilt values in these two directions, respectively, with positive values for the north and east tilts. Assuming $\varphi_{1}=0,\;\varphi_{2}$ = $\frac{\pi }{2}$, Equations (4) and (5) yield:

(6) $$\left\{\begin{array}{c} \xi =\theta \cos\psi \\ \eta =\theta \sin\psi \end{array}\right.$$

According to Equation (6), the following can be obtained:

(7) $$\left\{\begin{array}{c} \theta =\sqrt{\xi^{2}+\eta^{2}}\\ \psi =\tan^{-1}\frac{\eta }{\xi } \end{array}\right.$$

**Figure 4:**
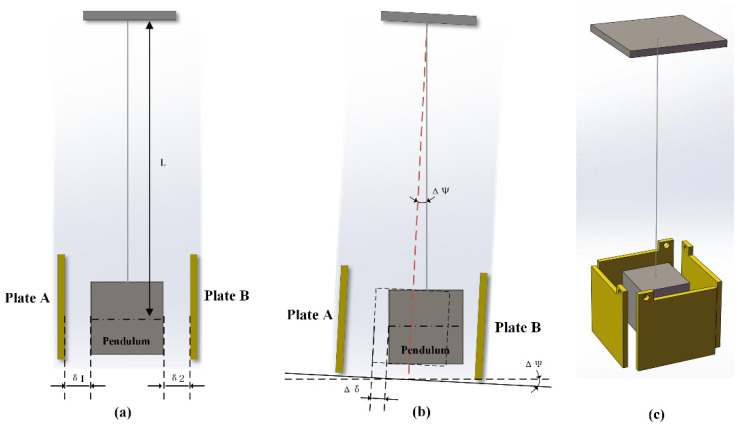
Schematic diagram of a vertical pendulum tilt sensor. Note: (**a**) Schematic diagram of the non-tilted condition. (**b**) Schematic diagram of the inclined state. (**c**) Schematic diagram of the three-dimensional structure of the pendulum body.

The vertical pendulum tilt sensor utilizes the formula’s principle to hang a gravity pendulum vertically on a stable support. The gravity pendulum M remains in a vertical position under the influence of Earth’s gravity. Assuming the original position of the pendulum is shown in Figure 4a when the ground tilts by an angle ΔΦ, the support experiences a corresponding tilt of ΔΦ, causing the pendulum M to undergo a relative displacement with the support as shown in Figure 4b. Δδ represents the displacement between the pendulum and the support caused by the ground tilt angle ΔΦ. The displacement of the pendulum is directly proportional to the ground tilt angle, enabling the measurement of small displacements to determine the tilt angle.

The ICT tiltmeter utilizes a single-suspension wire pendulum, as shown in Figure 4c. A capacitor pole plate is mounted on the front, back, left and right side of the pendulum. The effective spacing between the two sets of electrode plates, when the pendulum is removed, is fixed at 0.2 mm. The two sets of electrode plates share the pendulum as the intermediate electrode plate of a differential capacitor, allowing simultaneous measurement of the tilt in two orthogonal directions. The angle and direction of ground tilt are obtained through vector synthesis.

### 2.2. Design of a Digital Circuit for an FPGA-Based Vertical Pendulum Tiltmeter

**Figure 5:**
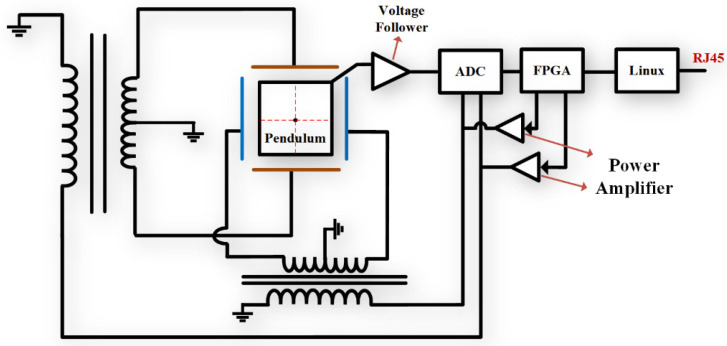
Measurement circuit of a high-precision component-type vertical pendulum tiltmeter based on an FPGA.

As shown in Figure 5, the FPGA control of high-speed ADC on the excitation signal and bridge output signal sampling, and do phase shift, phase sensitive detector, low-pass filtering and other digital signal processing, instead of the traditional analog circuitry, reducing the interference introduced by the active components, improve the resolution of the system. According to the different ground pulsation noise, the online configuration of the filter constants of sampling frequency and phase shift angle are more flexible to adapt to a variety of measurement needs, thus improving the efficiency of the use of equipment.

#### 2.2.1. FPGA High-Speed ADC Sampling and Digitization

High-speed and high-precision ADC sampling is essential for FPGA digital signal processing. The ADC of this system needs to sample the two excitation signals and the corresponding bridge unbalanced signal at high speed. The bridge unbalanced signal is stored and sent by the embedded system after digital signal processing, such as phase-sensitive detector and low-pass filtering through FPGA.

The system uses TI (TEXAS INSTRUMENTS) ADS1256 chip for analog-to-digital conversion; ADS1256 is a low-noise 24-bit analog-to-digital converter (ADC), and the maximum sampling rate of 30 Ksps. The ADC can communicate data with the FPGA through an internal SPI interface with an output format of 24-bit complement and a transfer rate of up to 1.5 Mbps [19,20].

**Figure 6:**
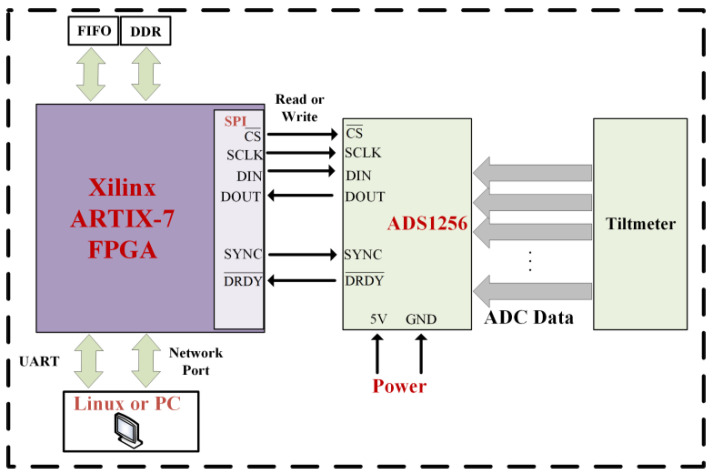
FPGA and ADC control block diagram.

**Figure 7:**
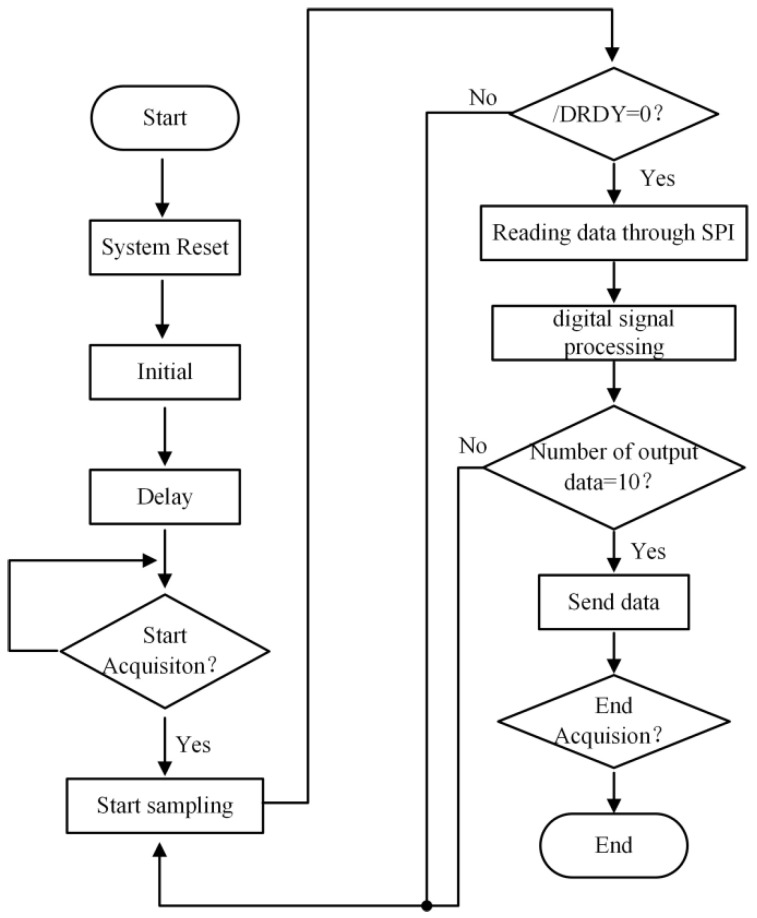
FPGA and ADC acquisition and control flowchart.

The FPGA chip used in this system is Xilinx’s A7 series chip XC7A35TFGG484, which has a six-input lookup table with 20 K, 250 user IO pins, 90 embedded hardware multipliers, etc., which is rich in resources and meets the requirements of this system. Verilog HDL programming is used to achieve the sampling of tilt data, FPGA signal processing, and storage and transmission [21]. The system connection and ADC acquisition flowchart are shown in Figure 6 and Figure 7.

The specific flow of the ADC acquisition part is as follows: after power-on, first carry out the reset, initialization and other operations, then determine whether to start the acquisition. If so, turn on the sampling enabler; when the/DRDY signal is low, it indicates that the current acquisition is complete. Read out the data through the SPI interface and carry out the digital signal processing, and after completing the processing 10 times, the data is sent through the network port or the serial port, and the Baud rate of the serial port adopted by the system is 115,200 bps [22,23].

#### 2.2.2. FPGA Digital Signal Processing

The pendulum serves as the intermediate electrode plate of the differential capacitor shared by the two bridges in the tilt measurement circuit, which uses two bridge circuits with excitation sources that have different frequencies (frequency doubling relationship). When the pendulum swings and produces displacement, the unbalanced signals of the two bridges are superimposed on the pendulum and then output via the signal-following circuit. The high-speed ADC samples the excitation signal and unbalanced bridge signal, and then the FPGA performs digital signal processing, such as phase-sensitive detection and low-pass filtering on the signals [24].

**Figure 8:**
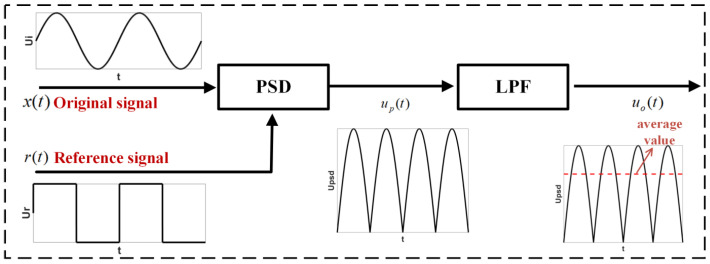
Schematic diagram of phase-sensitive detection operation.

The digital signal processing depicted in Figure 8 mainly consists of five parts: the unbalanced bridge signal (carrier), the reference signal (amplitude modulation wave), reference signal phase shifting, phase-sensitive detection (PSD), and low-pass filtering (LPF). Its core function is to convert the amplitude and polarity of the unbalanced bridge signal into a DC voltage output through phase-sensitive detection [25].

The phase-sensitive detector operation can be viewed as a multiplier, let the carrier signal be $x\left(t\right)=V_{s}(\cos\omega_{0}t+\theta )$, $V_{s}$ is the carrier amplitude. $\omega_{0}$ is the angular frequency, i.e., the excitation signal frequency. The reference signal is obtained by homologous excitation sampling and converted by the FPGA into a square wave with 50% duty cycle and ±1 amplitude. In other words, $r\left(t\right)=sign(\cos\omega_{0}t)$, and r(t) is expressed by the Fourier series in Equation (8) [26]:

(8) $$r\left(t\right)=\frac{4}{\pi }\sum_{n=1}^{\infty }\frac{{\left(-1\right)}^{n+1}}{2n-1}\cos\left[(2n-1)\omega_{0}t\right]$$

If there are interference signals of other frequencies in the carrier signal $V_{n}\cos\left(\omega_{n}t+\alpha \right)$, the carrier signal can be written as Equation (9):

(9) $$x\left(t\right)=V_{s}\cos\left(\omega_{0}t+\theta \right)+V_{n}\cos\left(\omega_{n}t+\alpha \right)$$

After phase-sensitive detection, the amplitude modulation square wave is multiplied by the carrier wave to obtain the detection signal $u\left(t\right)$, as shown in Equation (10):

(10) $$\begin{array}{c} u\left(t\right)=x\left(t\right)r\left(t\right)=\frac{2V_{s}}{\pi }\sum_{n=1}^{\infty }\frac{{\left(-1\right)}^{n+1}}{2n-1}\cos\left[\left(2n-2\right)\omega_{0}t-\theta \right]+\frac{2V_{s}}{\pi }\sum_{n=1}^{\infty }\frac{{\left(-1\right)}^{n+1}}{2n-1}\cos\left(2n\omega_{0}t+\theta \right)\;+\\ \frac{2V_{n}}{\pi }\sum_{n=1}^{\infty }\frac{{\left(-1\right)}^{n+1}}{2n-1}\cos\left[\omega_{n}t+\left(2n-1\right)\omega_{0}t+\alpha \right]+\frac{2V_{n}}{\pi }\sum_{n=1}^{\infty }\frac{{\left(-1\right)}^{n+1}}{2n-1}\cos\left[\omega_{n}t-\left(2n-1\right)\omega_{0}t+\alpha \right] \end{array}$$

The detection signal is then filtered by a low-pass filter to extract the carrier amplitude. The cutoff frequency of the low-pass filter is ω, where $\omega <<\omega_{0}$. Thus, when n ≥ 2, all terms in the above equation are AC components with frequencies greater than $\omega_{0}$, which will be filtered out after passing through the low-pass filter.

Furthermore, the terms in Equation (10) for *n* ≥ 2 are filtered out, and only the terms for *n* = 1 are retained. The detection signal can be expressed as Equation (11):

(11) $$u\left(t\right)=\frac{2V_{s}}{\pi }\cos\theta +\frac{2V_{s}}{\pi }\cos\left(2\omega_{0}t+\theta \right)+\frac{2V_{n}}{\pi }\cos\left[\left(\omega_{n}+\omega_{0})t+\alpha \right)\right]+\frac{2V_{n}}{\pi }\cos\left[\left(\omega_{n}-\omega_{0})t+\alpha \right)\right]$$

The second and third terms in the Equation will be filtered out after passing through a low-pass filter, leaving only the first and fourth terms. Then, $u\left(t\right)$ can be written as Equation (12):

(12) $$u\left(t\right)=\frac{2V_{s}}{\pi }\cos\theta +\frac{2V_{n}}{\pi }\cos\left[\left(\omega_{n}-\omega_{0})t+\alpha \right)\right]$$

It can be observed that the first term in Equation (12) represents the amplitude of the carrier signal. When θ=0, which means the amplitude modulation wave signal is in phase with the carrier signal, the detection effect is optimal. The second term represents the amplitude of the noise signal. Only when the noise frequency is very close to the carrier frequency, i.e., $\left|\omega_{n}-\omega_{0}\right|<\omega$, the noise be output together with the signal [21].

During actual operation, the FPGA performs phase shifting on the amplitude modulation wave through zero padding to match the phase of the carrier signal and achieve phase alignment. Therefore, the phase-sensitive detection circuit can be considered as a narrowband filter, allowing only the carrier with a frequency close to that of the modulation wave to pass through. In this system, the excitation signals of the two measurement bridges exhibit a frequency-doubling relationship. The unbalanced bridge signal superimposed on the pendulum can be separated and extracted after the respective amplitude modulation wave phase-sensitive detection and low-pass filtering without mutual interference.

#### 2.2.3. FPGA FIR Digital Filtering and Downsampling

In addition to filtering out the carrier and extracting the carrier amplitude, the low-pass filter also needs to remove environmental noise and comply with the sampling law to ensure that the output sampling satisfies the Nyquist theorem. The system utilizes a finite impulse response (FIR) digital filter for FPGA signal processing. The system function of the M-order FIR filter is represented by Equation (13):

(13) $$H(z)=\sum_{k=0}^{M-1}h(k)z^{-k}$$

This can be expressed by a difference equation where x(n) is the input sequence and y(n) is the output sequence, as shown in Equation (14):

(14) $$y(n)=\sum_{k=0}^{M-1}h(k)x(n-k)=h(0)x(n)+h(1)x(n-1)+\ldots +h(N-1)x(n-(N-1))$$

The system presented in this paper uses the Kaiser window function to design FIR low-pass filters [27]. The filter order is set to 15019, with a passband cutoff frequency of 0.025 Hz and a stopband cutoff frequency of 0.05 Hz. After quantizing the generated filter coefficients, a COE (Coefficient) file is generated and run on the FPGA using Xilinx Fir IP Kernel. The cutoff frequency of 0.025 Hz is chosen to meet the requirements of geophysical field tilt measurement and filter out ground microtremor noise.

**Figure 9:**
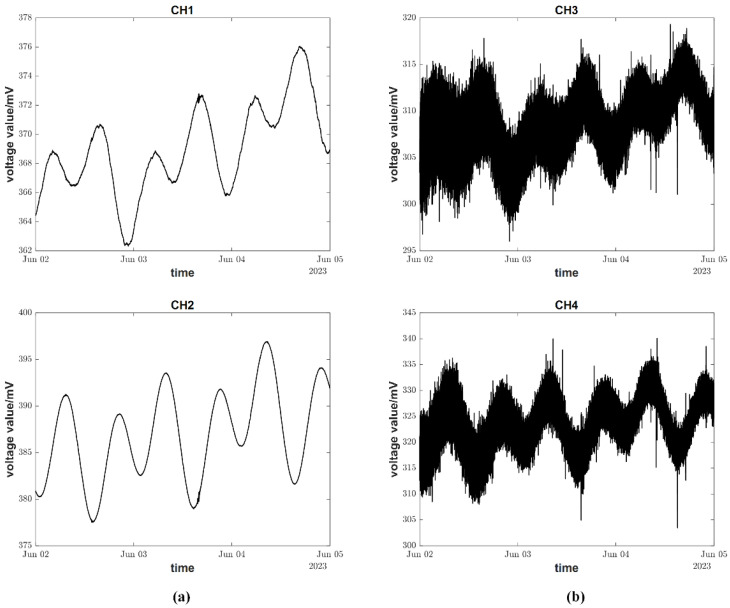
Ground tilt record curve. Note: (**a**) Filtered signal; (**b**) Signal before filtering.

For geophysical field deformation observation, a tilt measurement resolution of 0.2 mas (milliarcseconds) is required, and clear, solid tides are considered the standard for data evaluation. Since the amplitude of ground microtremor noise is similar to that of solid tides, filtering out ground microtremor noise is necessary to observe clear solid tides. Figure 9a shows the ground tilt measurement curve with a 0.025 Hz filter cutoff frequency, while Figure 9b shows the measurement curve with a 1 Hz filter cutoff frequency. It can be observed that during the same period of tiltmeter testing, the measured values with a 1 Hz filter cutoff frequency are mixed with excessive ground microtremor noise, resulting in unclear solid tide observations. However, the measured values with a 0.025 Hz filter cutoff frequency filter out most of the noise, enabling clear, solid tide observation with a high signal-to-noise ratio.

The frequency and amplitude of ground microtremor noise vary at different observation sites. Typically, low-pass filters have a cut-off frequency of 0.01–0.03 Hz, depending on the measurement requirements. Compared to analog circuits, FPGA-based signal processing allows for the flexible adjustment of the filter cutoff frequency, making it better suited to the measurement requirements of different stations and enhancing observation efficiency.

#### 2.2.4. FPGA Controlling the Excitation Source

In traditional single-ratio bridge measurement circuits, when the unbalanced bridge signal is too large, even if the signal is limited after passing through the following or amplifying circuit, the phase-sensitive detection curve remains in a half-wave shape since the signal’s fundamental frequency does not change. The detection curve still extracts the carrier amplitude after low-pass filtering even when it exceeds the limit, indicating the unbalanced degree in the bridge circuit. In this system, the pendulum serves as the intermediate electrode plate of a differential capacitor shared by two bridge circuits. The unbalanced signals of the two bridge circuits are superimposed on the pendulum, and the 2-channel signals are differentiated by their frequencies. However, when the tilt of the pendulum is too large, and the output of the signal-following circuit is limited, the original signal amplitude cannot be extracted after phase-sensitive detection and low-pass filtering.

**Figure 10:**
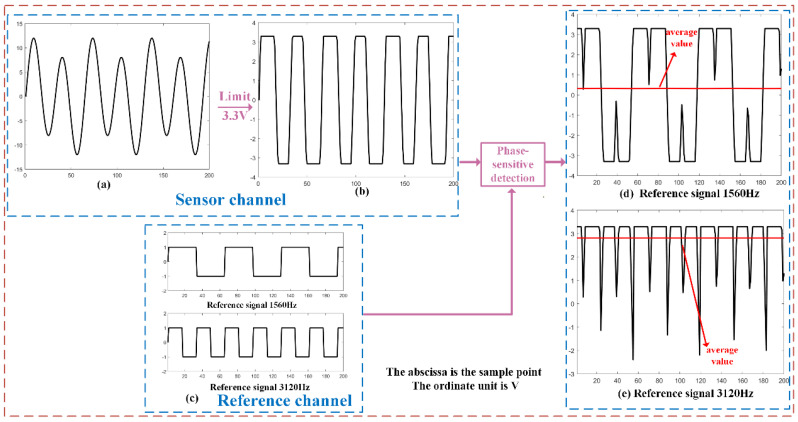
Schematic diagram of signal limiting and aliasing. Note: (**a**) Two synthesized signals. (**b**) Amplitude-limited signal. (**c**) Reference signal. (**d**) Phase-sensitive detector output signal when the reference signal is 1560 Hz. (**e**) Phase-sensitive detector output signal when the reference signal is 3120 Hz.

As shown in Figure 10, the power supply voltage of the signal-following circuit is ±3.3 V, meaning the output signal amplitude does not exceed ±3.3 V. The north–south tilt sensing signal frequency is 1.56 KHz with a signal amplitude of 10 V, and the east–west tilt sensing signal frequency is 781 Hz with a signal amplitude of 1 V. The two signals are synthesized, as shown in Figure 10a. After passing through the signal follower, the two-channel signals are limited and combined into the signal shown in Figure 10b, resulting in frequency aliasing of the signal. The superimposed signals are subjected to phase-sensitive detection at their respective frequencies, and the detection signals are shown in Figure 10d,e. It can be observed from the figures that the north–south and east–west signals, after low-pass filtering, have a value of 0.112 V and no longer accurately characterize the unbalanced degree of the corresponding bridge. Hence, when either or both signals are limited in amplitude, the final output voltage no longer clearly represents the tilt direction and magnitude. It may even exhibit a false zero phenomenon, where the output of the signal approaches 0 V when the pendulum swing is too large, and the signal is limited.

In this system, an FPGA generates a sinusoidal signal through DDS (Direct Digital Frequency Synthesis) as the excitation source, which then drives the ratio transformer via a power amplifier. By utilizing FPGA software to control the excitation source, the FPGA can halt the excitation of one channel in case of amplitude limiting, allowing the system to work in a single-channel measurement mode. Alternatively, it can lower the amplitude of the excitation signal to restore the unbalanced bridge signal within the non-limiting range, facilitating balance adjustment at a large tilt angle more conveniently and efficiently.

### 2.3. Design of Tilt Adjustment Triangular Platform

**Figure 11:**
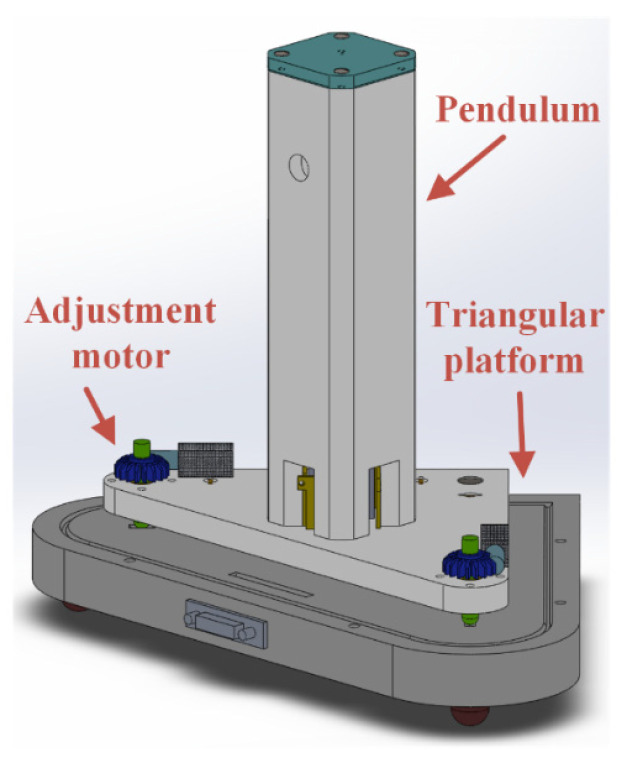
Schematic diagram of the tilting pendulum system and tilt adjustment triangular platform.

The structure of a capacitive vertical pendulum determines its limited dynamic range. In practical measurements, the pendulum requires a balance adjustment to position it at the center of the electrode plate. To facilitate this, capacitive vertical pendulum measuring instruments are typically installed on a tilt adjustment platform, allowing for a balance adjustment within the range of ±5°. In this system, the pendulum is mounted on a programmable, adjustable triangular platform, as shown in Figure 11 [28]. The triangular platform has a two-layer structure, with the lower bottom plate serving as a solid support foot that is directly connected to the ground. The upper platform holds the pendulum. The support screws of the upper platform are driven by a high-precision deceleration stepper motor controlled by a program, enabling precise adjustment of the upper platform within ±15°. Considering the driving accuracy of the stepper motor of 1 um, the adjustment platform can also serve as a calibration mechanism for the sensing unit, allowing for the online calibration of wide-range linearity and sensitivity [14,29].

To enhance the stability and impact resistance of the tiltmeter, a locking mechanism is installed on the upper part of the pendulum to push and lock the pendulum body. The entire pendulum body is also equipped with an outer sheath, and the pendulum system and upper platform are provided with sealing covers to ensure water and moisture resistance. This design allows the pendulum and balance adjustment motor to be used in harsh environments. When the pendulum system is locked and equipped with external packaging, it can meet the requirements of ordinary transportation and withstand a free-fall impact of 2 m, demonstrating high reliability.

## 3. Ground Tilt Observation

**Figure 12:**
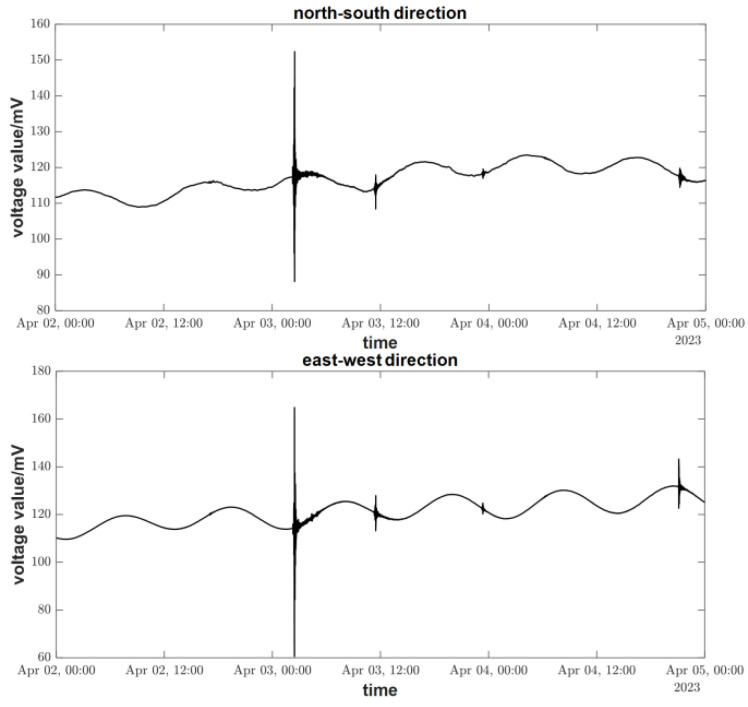
Tiltmeter measurement curve (2–4 April 2023).

The FPGA-based vertical pendulum tiltmeter was used for field observation at the seismic station chamber in Sixian County, Anhui Province, in January 2023. The chamber has a depth of 100 m and a relatively thick covering of over 30 m in the observation area. The annual average temperature change inside the observation cave is within 1 °C, providing a stable observation environment. The tiltmeter is installed in the side cave of the chamber, 60 m away from the entrance. A dedicated seismic monitoring pier is prepared to house monitoring equipment such as seismometers, gravimeters, and tiltmeters. The main parameters of interest in the geophysical field observations are solid tides and a co-seismic response. A solid tide refers to the phenomenon of periodic deformation of the solid earth under the action of the gravitational tidal force of the sun and the moon. The period of a tidal wave is about 23 h and 48 min, and the frequency is very low, approximating to 0 Hz. After the installation of the tiltmeter, clear, solid earth tides are recorded. Figure 12 shows the solid tide curve recorded by the tiltmeter, and the co-seismic response recorded in the figure corresponds to a 7.1-magnitude earthquake that occurred in Papua New Guinea on 3 April 2023 (4.25° S, 143.1° E). Firstly, the moments of violent fluctuations of the curves are consistent with the moments of earthquakes, indicating that the instrument can accurately record the moments of earthquakes. Secondly, the clear solid tide, as well as the seismic response, can be seen on the curve, indicating that the observation performance of the instrument meets the requirements. The instrument has now been in stable operation for ten months.

### 3.1. Solid Tide Prediction of Tiltmeter Observation Resolution

**Figure 13:**
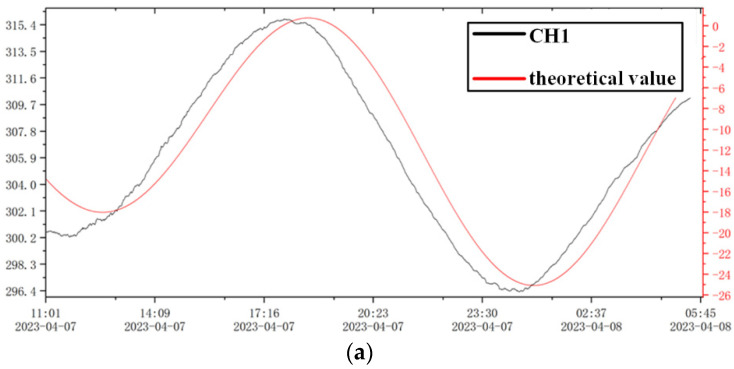

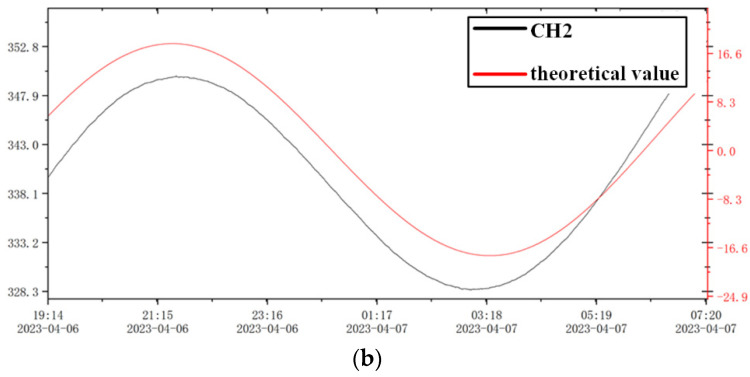
(**a**) Theoretical and observational curve of Sixian Station CH1 on 7 April 2023. (**b**) Theoretical and observational curve of Sixian Station CH2 on 7 April 2023.

The tiltmeter designed in this paper has a resolution of 0.2 mas (milliarcseconds) [13]; it is difficult to calibrate the instrument due to the difficulty of obtaining an accurate signal source in a laboratory setting. In geodetic and seismological research, the resolution of the instrument is typically obtained by comparing the measured solid tides by the tiltmeter with theoretical solid tides. Figure 13 shows the comparison of observed data on 7 April 2023 with theoretical solid tides. The resolution of the measurement system is calculated by comparing selected wave peaks or valleys. Following the testing method of the Technical Requirements of Instruments in Network for Earthquake Monitoring (DB/T31.2-2008) [30], the resolution of Sixian Station CH1 in the north–south direction is found to be 0.18 mas (milliarcseconds), and the resolution of CH2 in the east–west direction is 0.19 mas (milliarcseconds).

### 3.2. Calibration of Linearity and Sensitivity

The pendulum of the tiltmeter is mounted on a triangular platform, and the balance adjustment of the platform is driven by a stepper motor. The balance adjustment motor provides precise angle adjustment, serving not only as a zero-setting component but also as a calibration mechanism for the pendulum. During calibration, commands are sent through the network to drive the zero-setting motor with a certain step size, allowing calibration of the wide-range sensitivity, linearity, and scale value of the pendulum.

The following figure shows the complete record curve of the wide-range linearity calibration process. During calibration, the motor takes 100 steps per stepping, which is equivalent to a platform angle tilt of 1074 mas (milliarcseconds).

**Figure 14:**
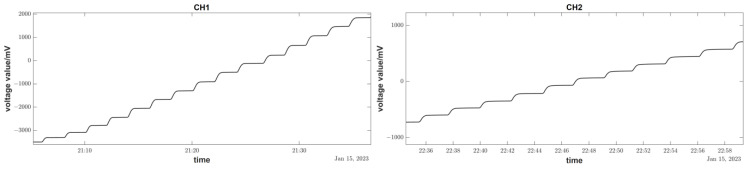
Calibration curve of wide-range linearity on-site.

According to the calibration curve in Figure 14, the linearity of the tilt sensors in channel 1 (north–south) and channel 2 (east–west) is 0.74% and 0.85%, respectively, meeting the calibration requirement of geophysical field tilt linearity of better than 1%.

## 4. Discussion and Conclusions

This paper introduces a high-precision component-type vertical pendulum tiltmeter based on an FPGA. By using a plumb-bob-type single-suspension wire vertical pendulum, it is capable of simultaneously measuring the ground tilt in two orthogonal components. The pendulum body is equipped with a locking motor and integrated sealing protection with the tilt adjustment platform, enhancing its reliability and impact resistance. The digital signal processing technology of FPGAs is widely employed in the system’s measurement circuits, replacing traditional analog circuits. This reduces hardware expenses and allows flexible online compilation and setting of measurement parameters, enabling adaptation to various measurement needs.

The field test at Sixian Station demonstrates that the introduced tiltmeter achieves a ground tilt observation resolution better than 0.2 mas (milliarcseconds), allowing for clear observation of solid tides and co-seismic responses. The harmonic analysis of the solid tide also meets the observation requirements of the geophysical field. During the test, the tiltmeter exhibits high reliability, strong adaptability, ease of use, and rapid deployment without the need for professional technical personnel. The system designed in this paper can be well applied in tilt observation; therefore, it holds great significance for geophysical field deformation observation.

## Data Availability

Not applicable.
